# Using participatory methods to design an mHealth intervention for a low income country, a case study in Chikwawa, Malawi

**DOI:** 10.1186/s12911-017-0485-6

**Published:** 2017-07-05

**Authors:** Rebecca Laidlaw, Diane Dixon, Tracy Morse, Tara K. Beattie, Save Kumwenda, Grant Mpemberera

**Affiliations:** 1 Department of Civil and Environmental Engineering, University of Strathclyde, Glasgow, UK; 2 School of Psychological Sciences and Health, University of Strathclyde, Glasgow, UK; 3 Department of Environmental Health, University of Malawi – the Polytechnic, Blantyre, Malawi; 4 Mfera Health Centre, Chikwawa, Malawi

**Keywords:** mHealth, Health education, Participatory, Contraception, Malawi

## Abstract

**Background:**

mHealth holds the potential to educate rural communities in developing countries such as Malawi, on issues which over-burdened and under staffed health centres do not have the facilities to address. Previous research provides support that mHealth could be used as a vehicle for health education campaigns at a community level; however the limited involvement of potential service users in the research process endangers both user engagement and intervention effectiveness.

**Methods:**

This two stage qualitative study used participatory action research to inform the design and development of an mHealth education intervention. First, secondary analysis of 108 focus groups (representing men, women, leadership, elderly and male and female youth) identified four topics where there was a perceived health education need. Second, 10 subsequent focus groups explored details of this perceived need and the acceptability and feasibility of mHealth implementation in Chikwawa, Malawi.

**Results:**

Stage 1 and Stage 2 informed the design of the intervention in terms of target population, intervention content, intervention delivery and the frequency and timing of the intervention. This has led to the design of an SMS intervention targeting adolescents with contraceptive education which they will receive three times per week at 4 pm and will be piloted in the next phase of this research.

**Conclusion:**

This study has used participatory methods to identify a need for contraception education in adolescents and inform intervention design. The focus group discussions informed practical considerations for intervention delivery, which has been significantly influenced by the high proportion of users who share mobile devices and the intervention has been designed to allow for message sharing as much as possible.

## Background

Mobile health (mHealth) is defined as “medical and public health practice supported by mobile devices such as mobile phones, personal digital assistants and other wireless devices” ([53, 54]; p. 6). mHealth applications can use both the phone’s basic functionalities (e.g. voice call and short messaging service) and more complex functionalities (e.g. third generation mobile telecommunications (3G) or Bluetooth technology). mHealth programmes cover a wide array of functions both in communicating with and monitoring the patient population, and for consultation, communication, training and development in healthcare professionals [55]. Types of programmes include SMS or telephone call appointment reminders for patients, mobile applications for continued education in healthcare workers and remote data collection for tracking and monitoring disease outbreaks [46]. One increasingly popular application for mHealth, particularly in low income countries, is its use in public health campaigns to provide preventative health education, due to its ability to increase participant reach in a relatively cheap and easy way [3, 5, 9]. For the purpose of this paper, mHealth refers to the use of mobile phones for preventative health education communication.

Mobile phone accessibility is imperative for mHealth interventions and ownership of mobile phones in low income countries has improved dramatically in recent years. In Malawi, 34% of the population own a personal mobile phone (29.1% in rural areas and 69.8% in urban areas), while 45.5% have a mobile phone in their household (40.2% in rural areas and 85.1% in urban areas) [36]. This is similar in Chikwawa, the study setting, where proportions of phone ownership in rural Southern Malawi, is 27.8% for individuals and 40.1% for households [36]. In addition, Malawians have the highest annual mobile phone expenditure (out of a 166 country study) at 56.6% Gross National Income per capita [21]. The cost of the device handset has been found to be this biggest barrier to mobile phone ownership, and 57.9% of individuals in rural Malawi are willing to pay between K2000-K5000 (equivalent of 3-7 US dollars) on a mobile handset [36], which would provide them with a basic mobile phone as smartphones are much more expensive. This is a substantial amount of money considering 87.6% of working adults in Malawi live on less than $3.10 per day [47]. From this evidence it would appear that those without mobile phones are willing to spend a significant proportion of their income on a mobile device, suggesting that mobile phone ownership is a priority to these individuals.

Health care in Malawi, although free, is poorly resourced, and hard to reach for many individuals, where they may face geographical barriers to access healthcare, poor infrastructure, and lack funds to travel [48]. Even when health care is accessed, Malawi has one of the highest doctor to patient ratios in the world at 0.019 physicians per 1000 people compared to 2.8 physicians in the UK [56]. The extensive staff and resource limitations in Malawian healthcare are underpinned by financial constraints, lack of trained workforce, and workforce migration [1, 41, 58]. This affects not only curative but also preventative health care, which if implemented effectively, could have a significant impact on reducing the main causes of morbidity and mortality e.g. HIV/AIDS and Malaria [57].

Health promotion is an integral part of the government’s healthcare strategic plan in Malawi [13], however with rapid increases in population growth [14] and the already overstretched healthcare system [52], more needs to be done to intervene in this sector, including the use of innovative ways to delivery health messages and achieve behaviour change. Therefore, with the ever increasing mobile phone usage, which is projected to double by 2020 [17], coupled with barriers in staffing and access to preventative healthcare, this low income country is a prime target for mHealth educational interventions.

mHealth interventions have the potential to overcome major problems in preventative healthcare delivery in developing countries. However, it is essential that their development and implementation are carried out with scientific rigour. Reviews of mHealth research both globally [7, 11, 45] and specific to developing countries [2, 18, 19] warn of the desperate need for adequately powered, rigorously tested and thoroughly evaluated scientific studies. In addition, intervention content must be tailored to the needs of the target population, for example, be sensitive to cultural context and demographic factors such as age and gender, in order to enhance intervention effectiveness [23]. Furthermore, participant level of understanding, language and literacy capabilities and technology skills also need to be considered [18]. Therefore participatory research, which involves all relevant stakeholders in intervention design and development, is imperative to increase the likelihood of intervention success and engagement [33].

Previous health education campaigns in Malawi, with a mobile component, have included ‘Chipatala Cha Pa Foni’ a hotline for expectant mothers [50] ‘Youth Alert!’ a reproductive health radio programme predominantly accessed via mobile phone [40], and health education ‘jingles’ via mobile phone platforms in the Millennium Village Project [30]. mHealth appears to be a popular mode of health education campaign delivery in Malawi. However peer reviewed research in Malawi has had a predominant focus on up-skilling community health workers to deliver messages [27, 29, 31], or implementing hotline or radio campaigns to specific groups of people ([39, 50]). There is little evidence regarding the impact of mhealth education campaigns at a community level.

Due to this gap in the current evidence base for community mHealth interventions, it is essential to conduct participatory action research to inform the design and development of an mHealth messaging service, which will provide health information to the community; this paper uses Chikwawa, Malawi as a case study. This two stage qualitative study involved local residents to identify health topic priorities and used these topics to inform a more detailed discussion of health education need, and the acceptability and feasibility of implementing an mHealth intervention in this area.

## Methods

### Study setting

**Fig. 1:**
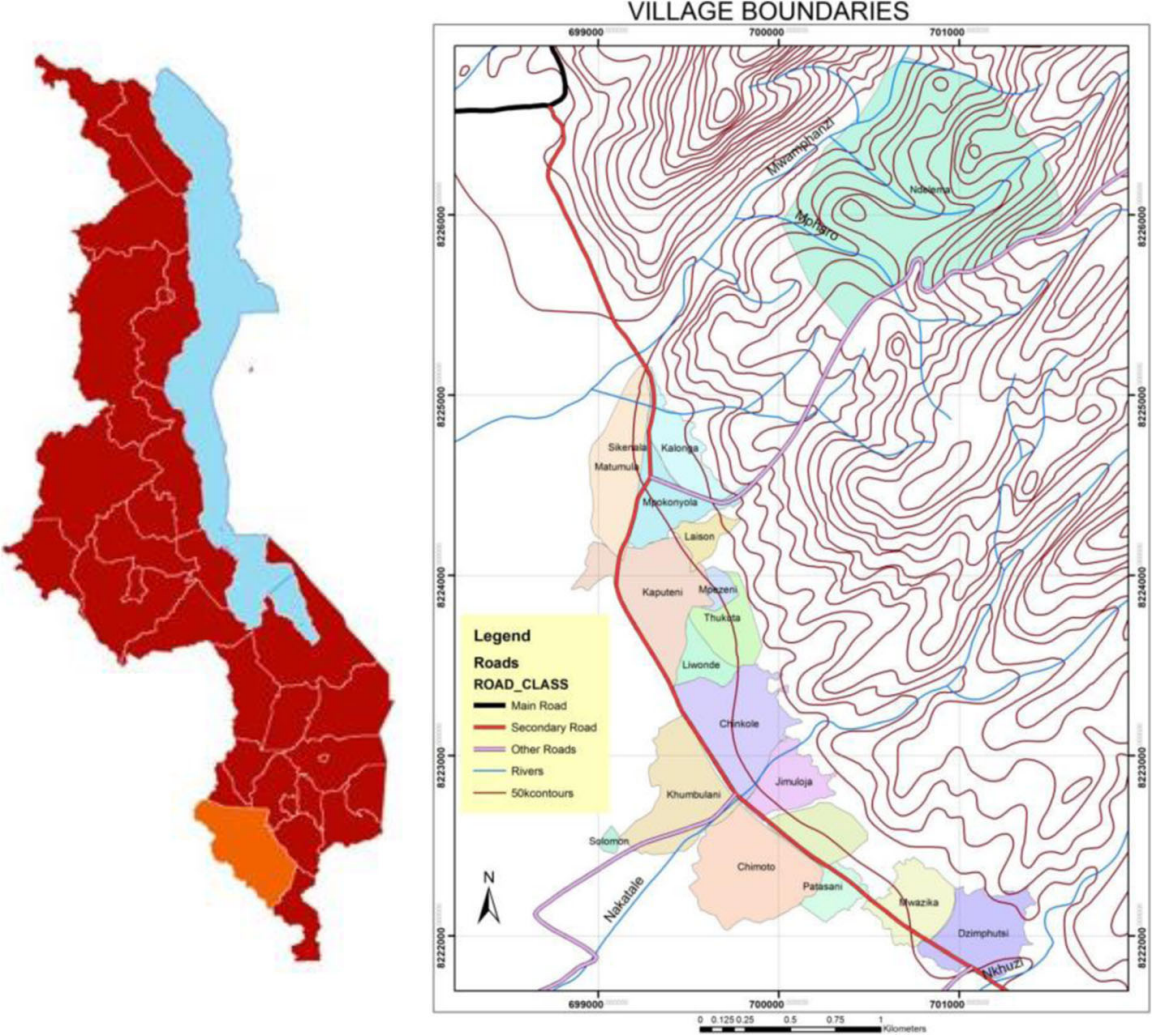
Map of Chikwawa District in Malawi and of the Study Setting. The maps were created using ArcGIS 10.2 software

This study occurred in the Mfera catchment area of Chikwawa, Malawi (Fig. 1). The research was integrated into the Scotland Chikwawa Health Initiative (SCHI), a consortium led by the University of Strathclyde; the initiative has implemented a variety of health improvement strategies in Chikwawa since 2006.

### Study design

The aim of the study was to conduct participatory action research to inform the design and development of a health education intervention, particularly intervention content, method of delivery and duration. This was achieved in two stages:

#### Stage 1: secondary analysis of village profiles

SCHI conducted 108 focus group discussions in 18 villages in Chikwawa (Fig. 1) in 2013/14. In each village, focus groups were conducted with 6 representative groups; Leadership (mixed gender), Men, Women, Elderly/Marginalised (mixed gender), Male Youth (15–24 years) and Female Youth (15–24 years), with group sizes ranging from 6 to 12. The data from each village was combined by a SCHI team member and summarised to form individual village profiles [43]. The 18 village profiles were used as secondary data to identify health education needs and the findings used to form the basis of Stage 2.

#### Stage 2: intervention development

Ten focus groups were conducted in two randomly selected villages, Sikenala (*n* = 42), Chimoto (*n* = 33) and one secondary school, Mfera Community Day Secondary School (CDSS) (*n* = 16) in 2015. Two focus groups were conducted with male adults, two with female adults, three with male youth and three with female youth. The demographic details of each group are displayed in Table 1.

**Table 1:** Demographics of 10 focus groups by sample population in Stage 2

Sample Population	Sample Size	Age Range (Years)	Literate (%)	Married (%)	With Children (%)	Education Level (mean number of years)
Male Adults	12	25–55	56	93	100	4.8
Female Adults	23	22–50	55	95	95	3.3
Male Youth	24	13–24	96	8	8	8.5
Female Youth	30	14–22	91	18	12	6.1

The study instrument was a series of open-ended questions informed by the findings in Stage 1. Questions focused on 4 health topics, Nutrition, Hygiene, Family Planning, HIV/AIDS, and included questions regarding mHealth intervention feasibility and acceptability. mHealth was presented to participants as the use of mobile phones to deliver health education via calls and text messages. Simple functionality was presented as observations in the communities indicated that the majority of residents only had access to basic mobile devices which could not access the internet. Questions addressed the relevance of each health topic, the perceived need for information about each topic, mobile phone ownership, intervention duration, mode of delivery and barriers to implementation. For adult focus groups there was additional discussion on parental consent for intervention access. Demographic information including age, literacy and education level was also recorded.

### Procedure

For both stages participants were recruited through convenience sampling. For community based focus groups, the facilitators walked around the village with the village Headman identifying eligible candidates. If participants agreed to take part, informed consent was obtained and members gathered at the meeting point for the focus groups to begin. At the secondary school participants were invited to take part by the Head teacher, and informed consent was obtained by facilitators prior to commencing the discussion. Focus groups were conducted in Chichewa by a trained facilitator and followed a pre-determined interview schedule. Additional probing questions were included to encourage expansion into discussion topics. Each focus group lasted between 45 and 60 min and were recorded using an audio recording device. All focus groups were transcribed verbatim and then translated into English by the facilitators.

### Analysis

#### Stage 1

Secondary analysis of the 18 village profiles recorded references to health education needs and these were tabulated by village and focus group type (leadership, men, women, elderly, male youth and female youth). To include observations made by the original interviewers and in instances where a health topic was discussed, but no identification of focus group was given, an extra group entitled ‘General’ was included in the analysis.

#### Stage 2

Thematic analysis was used to analyse the data, and followed the Braun & Clarke [4] step-by-step guide. This process included familiarisation with the transcripts and then highlighting sections relevant to the research question and assigning a descriptive label or code to these sections. Similar coded sections across all transcripts were collated to create themes. A discussion of themes was conducted and a consensus met as to which themes accurately represented the data collected.

## Results

### Stage 1

**Fig. 2:**
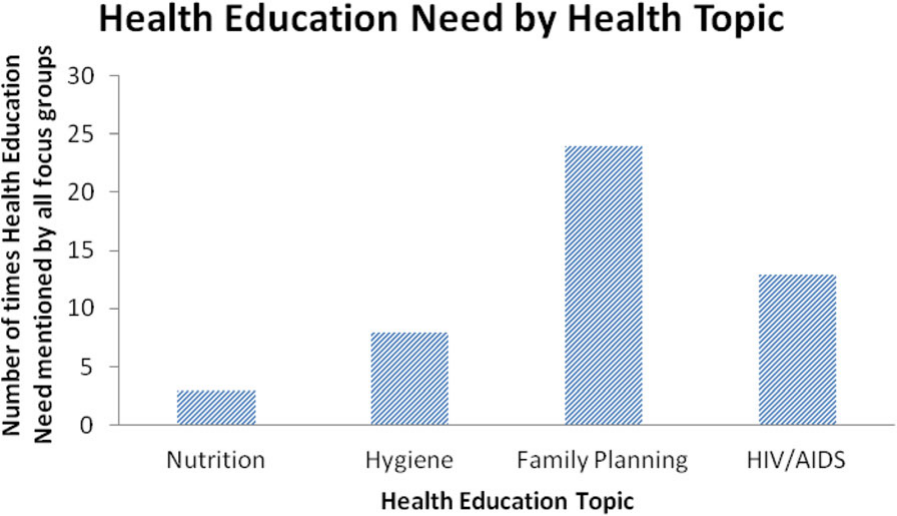
Health education need by health topic from secondary analysis of 18 village profiles in Stage 1

**Fig. 3:**
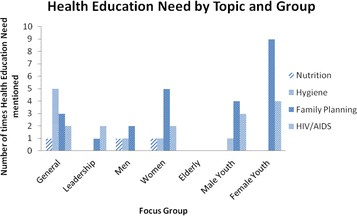
Health education need by health topic and represented population group from secondary analysis of village profiles in Stage 1

Fifteen of the village profiles referenced health education need in four health topics, Nutrition, Hygiene, Family Planning and HIV/AIDS (Fig. 2). Family planning was most frequently raised as a need, followed by HIV/AIDS. Figure 3 shows the breakdown of health education need by focus group, showing a clear expression of need in female youth, women and male youth for family planning education. Male and female youth account for 44% of the references for all health education need (combined adults account for 27%, general 23%, leadership >1%, elderly 0%) and were especially vocal in their need for youth friendly services, particularly surrounding family planning and HIV. For this reason youth were chosen by the researchers as the target group for the intervention.

### Stage 2

Ten focus groups were facilitated, with a total of 91 participants (Table 1).

Five themes emerged from the transcripts encapsulating opinions and beliefs of the participants around the four health topics.

### Recognised need of health education

This theme depicts participants’ self-reported need for health education and their prior knowledge of each health topic.

#### Need for health education

All focus groups recognised their need for health education. Amongst the four topics discussed, nutrition, hygiene, family planning and HIV, there was high concordance with the expressed health education needs at stage 1, with 80, 60, 100 and 90% of the groups describing a lack of information in these areas respectively. In six of the 10 focus groups participants highlighted a need to educate men on family planning methods. One female adult group and three focus groups with young people reported a need to increase knowledge of contraception methods in men, as it was suggested men do not always agree with their use. Similarly, two male adult and three focus groups with young people reported a need for men to be educated so that the decision on contraception uptake can be a joint one;

Male Adult, Sikenala: Contraception methods are perceived as for women in this village so if we receive these text messages in our homes we will be able to make an informed decision on which contraceptive method to use as a family.

This concurs with the Stage 1 analysis, where four male youth and two male adult focus groups raised the need for family planning education for men.

Participants expressed more specific health education needs in relation to family planning than any other health issue discussed (see Table 2). Furthermore, the perceived family planning needs showed a high degree of consistency across all four represented populations.

**Table 2:** Specific requests for Health Education in four health topics in Stage 2 (MA denotes Male Adults, FA - Female Adults, MY - Male Youth, FY - Female Youth)

Nutrition	Hygiene	Family Planning	HIV
Food groups [MA, FA, FY]	Hand Hygiene [MA, MY, FY]	Advantages to Family Planning [FA, MY, FY]	Prevention [MA,FA, MY, FY]
How to prepare a balanced meal [MA, MY, FY]	Toilet Practices^a^ [MA, FA, MY]	Inclusion of Men in Family Planning Education [MA, FA, MY, FY]	HIV Testing [MY, FY]
Nutrition for people living with HIV [MA]	Kitchen Hygiene [MA, MY, FY]	Contraception methods and side effects [MA, FA, MY, FY]	Treatment [MA, FY]
	Personal Hygiene^b^ [MA, FY]	Child Spacing [MA, FA, MY, FY]	
	Disease prevention [MA, MY]	STI’s [MY, FY]	
		Safe Motherhood/Early Pregnancy [FA, MY]	

^a^Toilet practices examples include washing hands after use, keeping the toilet clean and not practicing open defecation

^b^Personal hygiene examples include taking a daily bath and general cleanliness

#### Prior knowledge

Although there was high agreement about the need for health education in each topic, participants did demonstrate some prior knowledge. This was particularly the case in the areas of nutrition and hygiene, with seven groups able to describe the six food groups and eight groups able to explain toilet and household hygiene practices. There was less detailed knowledge of family planning and HIV. All of the focus groups mentioned the need for education on contraceptive methods; however only half of the groups demonstrated any knowledge about specific methods. The three methods that were discussed by these groups were Norplant (contraceptive implant), contraceptive injection and condoms. Interestingly, out of the four focus groups who mentioned Norplant and the injection, three stated that they knew the name but did not understand how the method worked or why it benefited them over any other method;

Female Adult Sikenala: We usually go for the injection but some people say it is not good for our health…we do not know the advantages and disadvantages of these contraceptive methods…we use any of the methods without really knowing the side effects.

Participants were able to list a variety of HIV related information requirements. Whilst this indicated some knowledge of HIV, participants had poor general understanding of HIV except for the link between condom use and prevention.

### Acceptability of an mHealth Intervention

This theme represents participants’ views on the acceptability of an mHealth intervention including message type, message volume and frequency.

#### Message delivery

Participants were asked their opinion of receiving health information on their mobile phone through a Short Message Service (SMS) text format or through a voice call format, Interactive Voice Response (IVR). Eight focus groups noted a preference to receiving this information through SMS;

Male Youth CDSS: The text messages because you can read them several times while a call you can get the health messages once.

Female Youth, Sikenala: …because we can read the messages while in automated calls you can miss the instructions you are being told.

Male Adults, Sikenala: …you can read a text message whilst doing other things right at home.

Participants felt that written texts were advantageous as the messages could be stored and referred back to whenever convenient and without time pressures of a one off call format.

However, two focus groups noted a preference for IVR technology over SMS;

Female Youth, Chimoto: Some of us prefer voice messages since some may not know how to read.

Both focus groups referred to IVR due to illiteracy levels and therefore this method would allow them to receive the health education.

Participants were also asked whether they would prefer to receive messages at set times (1 way messaging) or to access the information at their leisure through a text and reply service (2 way messaging). Male and female adults (4 focus groups) stated a preference for 1 way messages due to the messages being sent at fixed times. This appears to be due, in part, to difficulties surrounding the use of a mobile phone;

Female Adults, Chimoto: Do it at set times to help those who feel they cannot use a phone confidently.

Confidence in using a mobile phone, and technical ability is imperative to gaining access to mHealth education. Both male and female adults mentioned a lack of confidence in their own ability to use a mobile phone and they believe knowing in advance when the messages would come would help them. Young people did not mention user confidence as an issue; however 4 focus groups also noted a preference for getting the messages at set times;

Male Youth, Sikenala: We should receive notice of when the messages will come so that we should easily remember to go and read them.

Reliance on shared mobile phones was mentioned throughout the discussions and appears to influence preferences for a one way messaging service. Prior notice of when the messages would be delivered would allow for the individual to plan access to the device. The two final focus groups discussed both methods and were happy to receive messages at set times or at their leisure.

#### Time/frequency

Participants were asked to identify their preferred time and frequency of message delivery. While all ten focus groups were in agreement that they supported this method of health education delivery and would be willing to receive messages, there was no such agreement on specific timings for message delivery. Four focus groups (two female adult groups, female youth and male adults) stated that they had no preference;

Female Youth, Sikenala:…to some of us it doesn’t matter how many times we receive the messages, as long as we get the information.

Male Adults: Sikenala: the time doesn’t matter…even if you receive a message when you are asleep you can always check the message when you wake up.

A further four focus groups (two male youth groups, female youth and male adults) stated a preference for the afternoon, one stating 4 pm and the other 6 pm (two didn’t state a time). The final two focus groups requested messages to be sent out with school hours;

Male Youth, CDSS: You should send the messages after school hour; you should make sure they don’t affect our studies.

In addition to being out with school hours, participants also noted the importance of message timing in shared phone access, requesting messages to be sent at a time convenient for message sharing;

Female Youth, CDSS: …12 midnight can’t work because we will use borrowed phones.

Participants were also asked as to the frequency of message delivery. Four focus groups (representing each sample population) requested the messages to be sent at least twice per week, two focus groups (male and female youth) opted for three times per week, and two focus groups (female youth and female adults) for five times per week. The final two focus groups (male youth and male adults) did not state a preference and were willing to leave it up to the researchers;

Male Adults, Sikenala: …it’s up to you to decide that, we will always receive the messages.

### Barriers to phone access

Access to mobile phones to receive the health messages was a major discussion point in all focus groups, primarily barriers preventing access, including parental consent, mobile phone ownership and mobile sharing.

#### Phone ownership

Participants who owned a mobile phone were all in favour of the intervention and stated they would use the service upon implementation; this was however, only 24% of the sample. For those with only shared access to a mobile (60%), concerns arose around message service access;

Male Youth CDSS: …for us who use borrowed phones it won’t be easy to access the messages sometimes because the owners of the phones may be away.

Participants without mobile ownership have little control of when they get access to the phone. This may prevent consistent access to intervention content and is a significant barrier to intervention accessibility.

Participants were asked if they felt comfortable receiving health information through a phone which they did not own. Adult participants did not appear to find this an issue and were willing to use a shared phone;

Female Adults, Sikenala: we are all close so when one of us gets the message she would communicate…yes even if sharing husband’s phone that is no problem.

Only one male youth expressed apprehension regarding the sensitive content which would be delivered to a shared phone;

Male Youth, Chimoto: …to us who share with our relatives, we may not be that comfortable receiving reproductive health on a shared phone.

#### Parental consent

Young people were vocal in their need for health education, especially regarding reproductive health. Focusing the intervention on 15–24 year olds, requires parental consent for those under 16, therefore adult participants were asked their views on providing adolescents with this information;

Facilitator: What do you think of adolescents receiving this information on their mobile phone?

Male Adult, Chimoto: With this generation, it really has to be so.

All adult focus groups agreed to the need for adolescents to receive preventative health information, and stated they would not prevent their own children from accessing the messages.

In contrast, two female youth focus groups and one male youth focus group showed concern towards their parents’ reaction to their involvement in the study, especially if they were to access sensitive information on their parents’ phone.

Female Youth CDSS: …for me the parents would not like the [family planning] messages they would think that it’s a boyfriend giving me some advice.

Facilitator: Is it because you use a borrowed phone so you are afraid?

Male Youth, CDSS: Yes sometimes the parents may not be that friendly.

### Trust

This theme encompasses participants’ expectations of an mHealth intervention.

#### Involvement in Intervention Design

All focus groups stated that they would trust the messages they were given, and this appears to be due, in part, to their involvement in the process of intervention development;

Male Youth, Chimoto: Yes we will, because what we are discussing here is exactly what we want so we will trust you to give us the right messages.

Participants understood that their suggestions for health topics could become the basis for the message content, and liked that they were consulted about the practical issues surrounding the mHealth intervention. This appears to have positively impacted their perceived trust of the service.

Practically, it was noted that message content would be trusted if it was clear where the messages came from;

Female Youth, Sikenala: Yes, we will believe after seeing the phone number of the sender.

Facilitator: So that means we should have a unique number for project?

Female Youth Sikenala: Yes.

Belief in message content appears to stem from the trustworthiness of the sender. Participants stated they would believe the message content if they could be assured the messages came from SCHI through an identifiable phone number or short code.

#### Prior knowledge of service

In addition to recognising the sender, participants believed that prior knowledge of the service would impact their belief in the content;

Female Youth CDSS …only the people who are here would trust the messages because we would know it’s you when you send the messages.

Therefore it is perceived that involvement in this study will increase the likelihood of participants trusting the SMS message content.

### Sustainability

Sustainability refers to the participants views on the long term implication of the messaging service.

#### Follow up

Three focus groups expressed their desire to be involved in the development of the service and also expressed concern regarding intervention implementation;

Male Adult Sikenala: …plead to the authorities that they should not just make false promises but launch this programme.

This emphasises that participants are eager to see the SMS messaging service implemented in their area, and wish the project to provide regular updates of the research progress.

#### Face to face communication

Five focus groups (female adults, male youth and both male adult groups) requested face to face communication in addition to the messaging service;

Male Youth, Chimoto: …it will really be helpful if we had a club where the youth can meet and discuss about HIV and family planning.

Female Adult Sikenala: …apart from sending the messages you should come to conduct awareness campaigns to encourage us to use the health information.

This was to provide opportunities to discuss message content and to act as a reminder for continued use of the messaging service.

### Intervention development

**Fig. 4:**
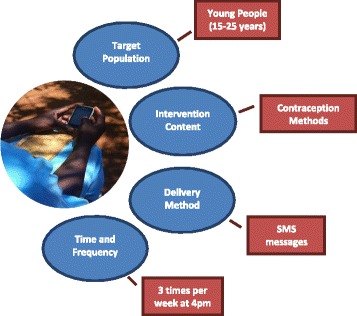
mHealth intervention development from focus group themes

Themes from Stage 2 informed the design and development of the mHealth intervention in terms of target population, intervention content, delivery method, and time and frequency of the intervention (Fig. 4).

## Discussion

This aim of this paper was to use participatory action research to inform the design and development of an mHealth preventative health intervention, as it is essential to tailor the intervention to participants needs to increase effectiveness in bringing about increased knowledge and behaviour change [19, 23].

### Target population

Of the four health education need topics (Nutrition, Hygiene, Family Planning and HIV) identified in Stage 1 and taken forward for further discussion in Stage 2, male and female youth accounted for the majority (44%) of references, and were particularly vocal about their need for youth friendly health services (YFHS) in Chikwawa. Providing access to YFHS has been a priority for the Government of Malawi [20], however accessibility to these services is challenging for young people and attendance is poor [10].

Previous research globally has targeted mHealth interventions towards young people to increase engagement with health services and introduce healthcare delivery via technology that is familiar and accessible to them [12, 38]. These studies have found promising results, with adolescents actively participating in pilot interventions delivering sexual health information and showing increased awareness of sexual health issues [28]. mHealth interventions therefore provide an attractive, novel and engaging method of information provision which could be used to provide adolescents with a tailored preventative health service, relevant to their needs. Although adults also expressed need for health education (27% of the references), the great need identified among youth for services specific to them and the technological appeal this mode of delivery has among this group were the key drivers in selecting young people as the target population for this intervention. This will support local YFHS in the area without adding extra pressure to overburdened local health centres that are not sufficiently staffed to run face-to-face interventions to this population group.

### Intervention content

Participants cited the need for more knowledge in each of the four health topics discussed in Stage 2. However, this was especially the case for family planning as all ten focus groups expressed a need for this information. This finding is in line with data from Stage 1, where family planning was also the most frequently requested topic, particularly by young people and women. These findings guided the decision to develop an mHealth intervention targeted towards family planning, where messages will describe contraceptive methods, their benefits and side effects, and where they can be obtained in relation to the study site. Family planning is a high priority in Malawi, and in line with Malawi’s Health Promotion Policy [15], as the consequences of unsafe sex are contributing significantly to the burden of disease in the country. In addition, reducing fertility rates due to the rapid increase in population is another government priority; the national population is estimated to triple by 2040 if fertility rates continue [14]. Therefore contraception education is an urgent necessity to help slow this rapid population growth.

Additionally teenage pregnancy is common in Malawi, [35] and this often leads to poor educational attainment in teenage girls, as they are forced out of school and seldom return [34]. It is imperative for family planning education to be provided to these teenage girls so that they can make an informed decision. This is especially relevant due to current widespread myths surrounding modern contraceptive use in rural Malawi, particularly the belief that infertility is a consequence of contraceptive use [6]. The lack of preventative health education services lead to this misinformation spreading within communities and may influence decisions regarding family planning practice. Consequently, it is crucial to provide young people with the correct medical information from a source they trust. SCHI has been working in this area for over a decade and the current study found the project as a trusted source of information.

It is also important for this education to be delivered to adolescent boys, and this has been called for as a priority in low income countries [16]. Results of the current study concur with this, identifying a perceived need to encourage males to use contraception and be involved in the decision making process for contraception uptake. This has been recognised by previous research, and family planning interventions have been created to target males in particular [22, 24, 44]. The inclusion of male youth in this intervention is important, as although current long-acting contraceptives target women, improvement in continuous use will require joint decision making of both partners [51].

### Intervention delivery method

Participants were encouraged to state their preference in delivery method of the intervention, either SMS messages or using IVR. The majority of focus groups opted for SMS messages over IVR as they can be stored, read again and have no time pressure regarding message delivery. Previous research in Malawi has found preference for IVR when given the choice between the two [8]. However SMS messages were more likely to be successfully delivered due to the need for greater technical support and user participation involved with IVR [8]. IVR has been used to combat high illiteracy levels [42] and even though two focus groups stated a preference for IVR due to illiteracy, our target population for this intervention has literacy rates of 91% for female and 96% for male youth respectively. However this preference may be specific to this sample, and if the intervention was to be delivered on a widespread scale, the intervention delivery method would need to be continually reassessed to ensure equality of access for all.

In addition, participants repeatedly cited a desire for face-to-face communication, with some unsure if messages alone were enough. This is the first study looking at perceptions of mHealth in Chikwawa, Malawi; therefore lack of experience with SMS may have favoured their opinion towards face-to-face knowledge exchange which is the norm. Evidence shows mobile-based behaviour change interventions are more effective with additional delivery methods, i.e. mHealth plus face-to-face or telephone call [26]; however individual SMS interventions have also produced greater behaviour change than routine healthcare [37]. Therefore consideration for potential face-to-face additions to the intervention, particularly around the creation of youth groups at the local health centre will be assessed in the next phase of the research. This would provide a platform for peers to discuss the mobile content and ask questions to a trained facilitator.

### Timing and frequency of the intervention

Gurman et al. [18], in their review of mHealth behaviour change interventions, found that formative research into the preferred timing of health communication is seldom undertaken, yet important in order to understand the target population’s preferences. In addition, mHealth interventions will likely be more effective if message content, frequency and style are relevant to the needs and preferences of the target population [45].

Adults stated a preference for messages to be sent at set times (one-way messaging) due low user confidence in mobile phones, and the majority of youth noted a preference for receiving messages at set times due to the ability to plan shared access of mobile phones. Specific preferences for message frequency and timings were mixed in the sample with no majority for either. Message frequency suggestions ranged from twice per week to five times per week, and participants emphasised the need for messages to be delivered after school hours and at times which allowed for message sharing, with a consensus for afternoon hours. References to message sharing are important, as in this sample 24% of participants owned a mobile phone and 60% cited at least one relative or friend with ownership. This is in line with the national statistics stating household mobile phone access (45.5%) as higher than individual level access (34%) [36], The proportion of participants with frequent access to the shared or borrowed devices was unclear, however, there is potential to reach 84% of the participants sampled.

Due to the reliance on shared mobile phones, it is important to design the intervention to be as accessible as possible. Therefore the intervention will use a one-way messaging system to allow messages to be sent to everyone at the same time. SMS messages will be sent three times per week at 4 pm – to allow for shared phones to be accessed after school and before dark. The timings and frequency will be further evaluated and refined after the next phase of research to ensure it continues to suit the target population.

From this sample, the majority of participants would rely on others for information, so it is imperative to prioritise ways to encourage phone owners to share message content. This will be considered in the design of the intervention, and will include informing the owner of shared devices about the intervention during recruitment, to increase likelihood of participants receiving permission to borrow the device. There is no publication known to the authors addressing the use of shared mobile devices for sensitive health education such as contraception. However previous studies have identified SMS interventions for family planning education as an acceptable method in low income countries [25, 49], but do not separate opinion against phone access. The current study highlights that although adolescents worried about their own parents’ reaction to them receiving information on a sensitive health topic like contraception, all adult focus groups expressed the view that young people need to receive this information. Even though parents of the adolescent participants were not directly targeted, it appears adult participant who have adolescent children support the delivery method and educational content; the young people’s concerns about their parents’ perceptions may therefore be misplaced.

It was important to take into consideration the views of both adults and young people to ensure that both parties agree with the mHealth intervention, particularly in cases where adolescents may register their parents’ phone and a lack of inclusion in this study could make them wary of the research. This was identified in the current study, as involvement in the process of intervention development, and knowledge of its existence were stated as reasons for trust in the information received. Therefore involving parents at this stage creates awareness of the intervention, and as SCHI has a long standing relationship with residents in the area, will also provide reassurance as to the planned mHealth intervention.

### Intervention development

This paper has established that the sampled participants support the idea of an mHealth messaging service in Chikwawa and the discussions have the design of an intervention tailored to their needs. Messages will be 160 characters to adhere to the standard SMS limit for basic phones, allowing any type of phone to access the intervention content. The messages will be based on the WHO guidelines for family planning [53, 54] and include behaviour change techniques from the behaviour change taxonomy [32]. Intervention development will be in collaboration with both the Ministry of Health in Malawi and the local District Health Office to ensure the messages are in line with national health strategies. The next phase of research is to pilot the intervention in the community to determine the feasibility and acceptability. This work will particularly address user experience in shared mobile devices to access the intervention, assess willingness to attend youth clubs in the area in order to introduce a face to face element to the intervention, and evaluate local infrastructure for successful intervention delivery.

### Limitations

Due to the recruitment of participants being led by directions from village headman and the head teacher’s recommendations there is potential selection bias in the sample. However there is still a diverse age range and education level within the groups and care was taken to include students currently in education.

## Conclusion

mHealth has the potential to deliver fundamental preventative health messages to areas of the community who are difficult to reach, and which cannot be delivered by the current under-resourced and overstretched health facilities. This study has used participatory methods to identify a need for contraception education in adolescents and inform intervention design. This is both a national priority and a requirement in order to curb population growth, prevent teenage pregnancy and overcome myths and misinformation influencing the perceptions of contraceptive use in young people. The focus group discussions have also informed practical considerations for intervention delivery, which has been significantly influenced by the high proportion of users who share mobile devices and the intervention has been designed to allow for message sharing as much as possible. Encouraging sharing will not only increase education reach, but will provide a platform for open communication between parent and child, and within marriages, two important issues raised in the discussions. Efforts now need to be focused on ensuring continuous participant involvement in intervention development and evaluation in the feasibility phase of this research, as this is repeatedly cited in the literature as a necessity in intervention development, but also requested by the participants in this study.

## Abbreviations

CDSS: Mfera Day Secondary School
HIV: Human immunodeficiency virus
IVR: Interactive voice response
mHealth: Mobile health
SCHI: Scotland Chikwawa Health Initiative
SMS: Short message service
WHO: World Health Organisation
YFHS: Youth friendly health services
